# Age-Dependent Inflammatory Microenvironment Mediates Alveolar Regeneration

**DOI:** 10.3390/ijms25063476

**Published:** 2024-03-20

**Authors:** Rui Quan, Chenhong Shi, Bing Fang, Yanan Sun, Taiqi Qu, Xifan Wang, Ran Wang, Yiran Zhang, Fazheng Ren, Yixuan Li

**Affiliations:** 1 Key Laboratory of Precision Nutrition and Food Quality, Department of Nutrition and Health, China Agricultural University, Beijing 100083, China; ruiquan@cau.edu.cn (R.Q.); shichenhong@cau.edu.cn (C.S.); bingfang@cau.edu.cn (B.F.); syn612@cau.edu.cn (Y.S.); wangran@cau.edu.cn (R.W.); yiran1119@cau.edu.cn (Y.Z.); renfazheng@cau.edu.cn (F.R.); 2 Key Laboratory of Functional Dairy, Co-Constructed by Ministry of Education and Beijing Municipality, College of Food Science & Nutritional Engineering, China Agricultural University, Beijing 100083, China; qyqutaiqi1997@cau.edu.cn; 3 Department of Obstetrics and Gynecology, Columbia University, New York, NY 10032, USA; wangxfan@126.com

**Keywords:** proliferation, differentiation, inflammation, AT2, alveolar regeneration

## Abstract

Lung aging triggers the onset of various chronic lung diseases, with alveolar repair being a key focus for alleviating pulmonary conditions. The regeneration of epithelial structures, particularly the differentiation from type II alveolar epithelial (AT2) cells to type I alveolar epithelial (AT1) cells, serves as a prominent indicator of alveolar repair. Nonetheless, the precise role of aging in impeding alveolar regeneration and its underlying mechanism remain to be fully elucidated. Our study employed histological methods to examine lung aging effects on structural integrity and pathology. Lung aging led to alveolar collapse, disrupted epithelial structures, and inflammation. Additionally, a relative quantification analysis revealed age-related decline in AT1 and AT2 cells, along with reduced proliferation and differentiation capacities of AT2 cells. To elucidate the mechanisms underlying AT2 cell functional decline, we employed transcriptomic techniques and revealed a correlation between inflammatory factors and genes regulating proliferation and differentiation. Furthermore, a D-galactose-induced senescence model in A549 cells corroborated our omics experiments and confirmed inflammation-induced cell cycle arrest and a >30% reduction in proliferation/differentiation. Physiological aging-induced chronic inflammation impairs AT2 cell functions, hindering tissue repair and promoting lung disease progression. This study offers novel insights into chronic inflammation’s impact on stem cell-mediated alveolar regeneration.

## 1. Introduction

Chronic lung diseases have emerged as the third leading cause of mortality worldwide, with advancing age serving as a significant predisposing factor in the pathogenesis and progression of diverse pulmonary ailments [1,2]. Empirical evidence derived from epidemiological inquiries reveals an overwhelming predominance of pneumonia-related deaths among elderly individuals aged 65 years and older, accounting for approximately 85% of such cases. In contrast, the younger demographic below 45 years contributes a mere 3% [3]. Furthermore, research indicates that individuals aged 80 years and older infected with SARS-CoV-2 have a mortality rate over 20 times higher than their counterparts over 50 years old [4]. Evidently, the aging process results in a progressive deterioration in pulmonary functionality, increasing vulnerability to lung diseases while compromising the protective barriers of the lung tissue.

The lungs, being the largest organ by surface area in the human body [5], are critical for gas exchange. Lung function peaks between 18 and 25 years of age and remains stable until around 35 years old. However, subsequent to this period, the progressive aging process gives rise to noticeable alterations in the lungs’ tissue structure, gas exchange function, and immune response mechanisms [6]. The alveolus, the smallest unit responsible for gas exchange in the lungs, consists of a monolayer of epithelial cells. It serves as a critical site for gas exchange between inhaled air and circulating blood while acting as a protective barrier against harmful substances like pathogens, particulate matter, and other foreign materials [7]. However, with aging, the protective function of this pulmonary epithelial barrier gradually declines, resulting in reduced responsiveness to environmental stressors. Aging lung tissue exhibits progressive damage and the infiltration of inflammatory cells, accompanied by increased secretion of Senescence-Associated Secretory Phenotype (SASP) and inflammatory factors and the destruction of the alveolar structure. Therefore, effective alveolar regeneration to repair and maintain barrier integrity is crucial for preventing age-related lung diseases.

The epithelial structure is a crucial factor in maintaining alveolar regeneration. The alveolar epithelium principally comprises two distinct cell types: type I alveolar epithelial cells (AT1) and type II alveolar epithelial cells (AT2) [8]. AT1 cells, characterized by their large and flat squamous morphology, span more than 95% of the alveolar surface, providing an expansive area conducive to efficient gas exchange. Meanwhile, AT1 cells are also the most vulnerable target cells in lung injury and aging. Remarkably, AT1 cells themselves lack the capacity for proliferation. Conversely, AT2 cells serve as stem cells with differentiating capabilities, and their differentiation into AT1 cells is crucial for regenerating epithelial structures. Normally, the bulk of AT2 cells remain quiescent, exhibiting limited regenerative potential [9]. However, in response to injury signals, AT2 stem cells undergo differentiation and proliferation, ultimately transitioning into AT1 cells to restore the damaged alveolar epithelium [10]. Previous research revealed that in a bleomycin-induced lung injury model, 12-month-old mice exhibited more severe damage to the alveolar epithelium compared to 3-month-old mice, and during a 63-day observation period, their capacity for alveolar self-repair was weaker than that of the 3-month-old mice [11]. Aging-related apoptosis, depletion, and the dysfunction of AT2 cells directly impair lung repair and regeneration. The proliferation and differentiation of AT2 cells are critical indicators of alveolar epithelial repair and must be precisely regulated to maintain alveolar integrity and efficient repair [12,13]. The disruption of this equilibrium may result in grave pulmonary ailments. The robust vitality and differentiation capacity of AT2 cells are essential for maintaining a sufficient supply of AT1 cells.

As research on AT2 cells progresses, we are uncovering various factors that can impact their self-renewal and differentiation capabilities, including oxidative stress [14], telomere damage [15], and mechanical tension [16], among others. The influence of chronic inflammation resulting from natural aging on the stemness of AT2 cells is increasingly under scrutiny. Chronic inflammation is a persistent state of inflammation that causes tissue damage and may affect stem cell function through multiple pathways. However, it is currently unclear how aging reshapes the microenvironment of lung stem cells and whether the inflammation response induced by aging is a key factor in inhibiting the regeneration of epithelial structures.

In this study, we systematically studied dynamic changes in the quantity, structure, and function of alveolar epithelial cells from lung maturation to senescence, employing a naturally aging mouse model. Additionally, we utilized transcriptomics to further explore the potential factors influencing AT2 function, uncovering a correlation between inflammatory factors and the proliferative and differentiating capabilities of AT2 cells during the aging process. Our study contributes to a comprehensive understanding of the impact of inflammation on alveolar regeneration, offering new insights and directions for delaying or treating age-related chronic pulmonary diseases through systemic inflammation modulation strategies.

## 2. Results

### 2.1. During Lung Aging, the Alveolar Epithelial Layer Sustained Damage

**Figure 1:**
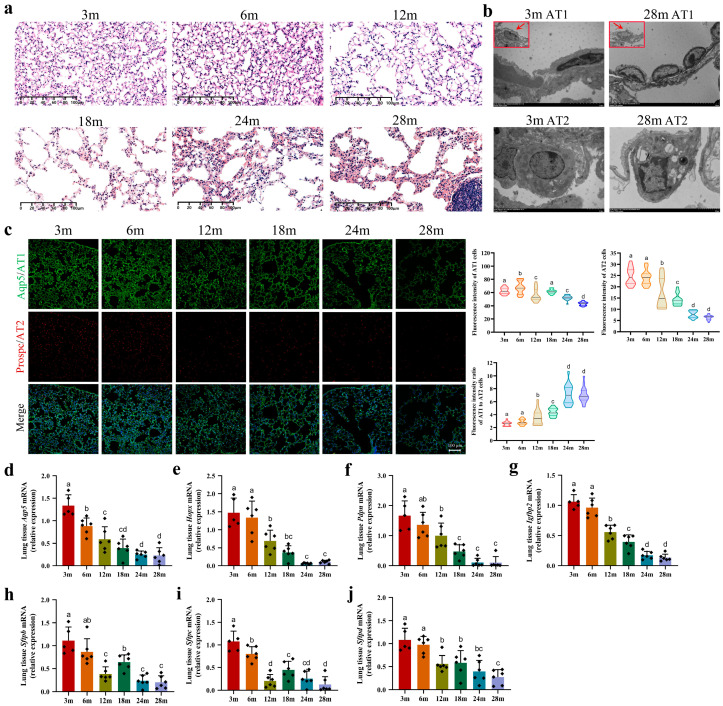

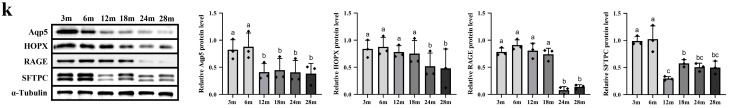
Age-related changes in lung structure and epithelial cell number. (**a**) Histological analysis of lung tissue from mice at different ages (scale bar: 100 μm), n = 6 mice; (**b**) high-resolution TEM imaging of AT1 (scale bar: 5 μm) and AT2 cells (scale bar: 2 μm) in 3 m and 28 m mice (n = 3); (**c**) immunofluorescence analysis of AT1 and AT2 cells in lung tissue (objective: ZEISS Plan-Apo 10X, 0.8NA). Violin plots show fluorescence intensity variations for AT1 and AT2 cells, as well as the AT1/AT2 ratio (n = 3 mice, with 6 quantified fields per tissue section); (**d**–**j**) qRT-PCR analysis of surface marker expression of (**d**) *Aqp5*, (**e**) *Hopx*, (**f**) *Pdpn*, and (**g**) *Igfbp2* in AT1 cells and (**h**) *Sftpb*, (**i**) *Sftpc*, and (**j**) *Sftpd* in AT2 cells in mouse lung tissues. Each group comprised 6 biological replicates (except for the 3 m group (n = 5), excluding an outlier data point from one mouse in the analysis), with 3 technical replicates. RT-qPCR results presented as mean ± SD values, with significant group differences denoted by lowercase letters (*p* < 0.05); (**k**) Western blot analysis of surface marker proteins Aqp5, HOPX, and RAGE in AT1 cells and SFTPC in AT2 cells in mouse lung tissues (n = 3), normalized to α-Tubulin loading control.

To investigate alveolar structural alterations, we conducted comprehensive histopathological examinations of lung lobes using HE staining. As shown in Figure 1a, young mice at 3 m and 6 m exhibited a greater abundance of intact alveoli and a more widespread distribution of alveolar epithelial cells. Conversely, adult mice at 12 m demonstrated significant reductions in the numbers of alveoli and alveolar epithelial cells. Certain alveolar walls ruptured, leading to the merging of adjacent alveoli into larger structures. In middle-aged mice at 18 m, pronounced alveolar collapse was observed, accompanied by a disrupted alveolar epithelium and thickened alveolar septa. Localized alveolar cavities exhibited mild infiltration of red blood cells and inflammatory cells. As the mice reached an elderly state at 24 m and 28 m, the structure of the alveolar epithelium became unrecognizable, with inflammatory cells, red blood cells, and fibrous exudates accumulating within the alveolar cavities, causing blockages or displaying cuff-like structures.

Next, we utilized a transmission electron microscope (TEM) to further examine the alterations in the microstructure of the alveolar epithelial layer during the aging process. Our observations revealed that the structure of the 3 m AT1 cells was intact, with clear cell nuclei, prominent nucleoli, numerous heterochromatin masses, and a clustered distribution (Figure 1b). However, the 28 m AT1 cells exhibited a soybean-shaped appearance with a smaller volume compared to the 3 m cells. Additionally, the cell nuclei became elliptical, the heterochromatin decreased, the cytoplasm became thinner, and the cytoplasmic structure was disrupted or even partially absent. In addition, we observed intact microvilli structures on the surfaces of 3 m AT2 cells, characterized by round cell nuclei and prominent nucleoli. The cytoplasm exhibited abundant mitochondria and onion-like lamellar bodies (Figure 1b), indicating that these cells are metabolically active, display vigorous growth, and are generally healthy. However, in the 28 m AT2 cells, the microvilli significantly decreased or disappeared. The overall cell structure became disorganized, with mostly emptied lamellar bodies and a notable reduction in cytoplasmic mitochondria. Additionally, aging AT2 cells exhibited irregularly shaped nuclei with condensed chromatin arranged along the nuclear membrane, forming blocks of various sizes. Furthermore, spherical myelin-like bodies appeared in the cytoplasm, potentially associated with cellular autophagy. In conclusion, age-related structural modifications in the alveolar epithelial layer inevitably impair the gas exchange function of the lungs.

### 2.2. The Number of Alveolar Epithelial Cells Decreased during the Lung Aging Process

In this study, we performed immunofluorescence staining using specific antibodies (Aqp5 and Prospc) to assess changes in the numbers of AT1 and AT2 cells during lung aging. The results from Figure 1c indicate a significant decrease in both the green fluorescence of AT1 cells and the red fluorescence of AT2 cells with aging. We utilized ImageJ software version 1.53 to measure the average fluorescence intensity of these cells within a defined area. Our analysis revealed that the number of alveolar epithelial cells remained stable at 3 m and 6 m of age. However, declines in the numbers of AT1 and AT2 cells were observed from 12 m to 28 m. Throughout the aging process (from 3 m to 28 m), the fluorescence intensity of the AT1 cells decreased by 28.1%, while the fluorescence intensity of the AT2 cells decreased by 72.2%. The ratio of AT1 fluorescence intensity to AT2 fluorescence intensity showed an increasing trend, with a 1.68% rise from 3 m to 28 m. These findings suggest that the decline in the number of AT2 cells outpaced that of the AT1 cells, directly impacting the regenerative capacity of the damaged alveoli.

Moreover, we quantified the mRNA levels of surface-specific proteins on alveolar epithelial cells to further evaluate the quantities of AT1 and AT2 cells. Our results revealed age-dependent declines in the expression levels of the proteins *Aqp5*, *Hopx*, *Pdpn*, and *Igfbp2* on the surfaces of AT1 cells (Figure 1d–g). As illustrated in the figures, a conspicuous discrepancy was discernible beginning from the 12 m group, with a substantial reduction in the expression of these genes relative to the 3 m group. This precipitous decline persisted until 24 m, whereupon it stabilized until 28 m. It is significant that a notable contrast was evident between the 24 m and 28 m groups compared to the 3 m group. An intriguing observation revealed a pronounced decline in the expression levels of the *Sftpb*, *Sftpc*, and *Sftpd* genes (which were highly expressed in AT2 cells from 3 m to 12 m). This trend experienced a temporary reversal at 18 m before transitioning to a sustained, age-dependent decrease from 18 m to 28 m (Figure 1h–j). Notably, a markedly significant disparity was evident between the 28 m group and the 3 m group. We further conducted protein-level detections of Aqp5, HOPX, and RAGE (AT1 cells), as well as SFTPC (AT2 cells), yielding similar results to those obtained at the mRNA level (Figure 1k).

### 2.3. The Self-Proliferative and Differentiating Capacities of Alveolar Epithelial Cells Declined during Lung Aging

**Figure 2:**
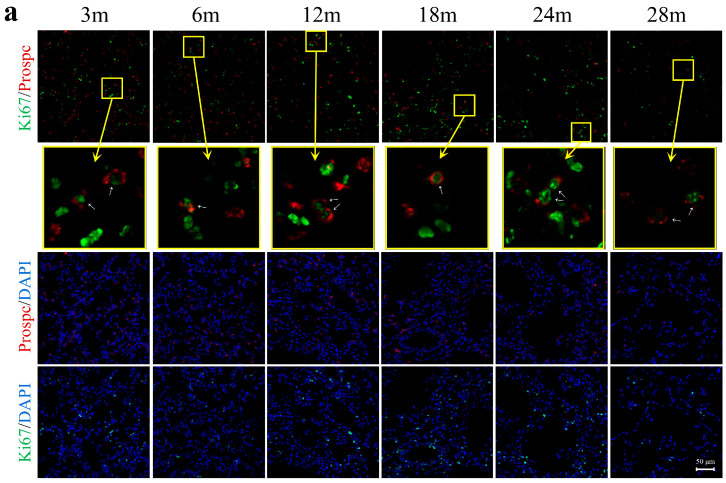

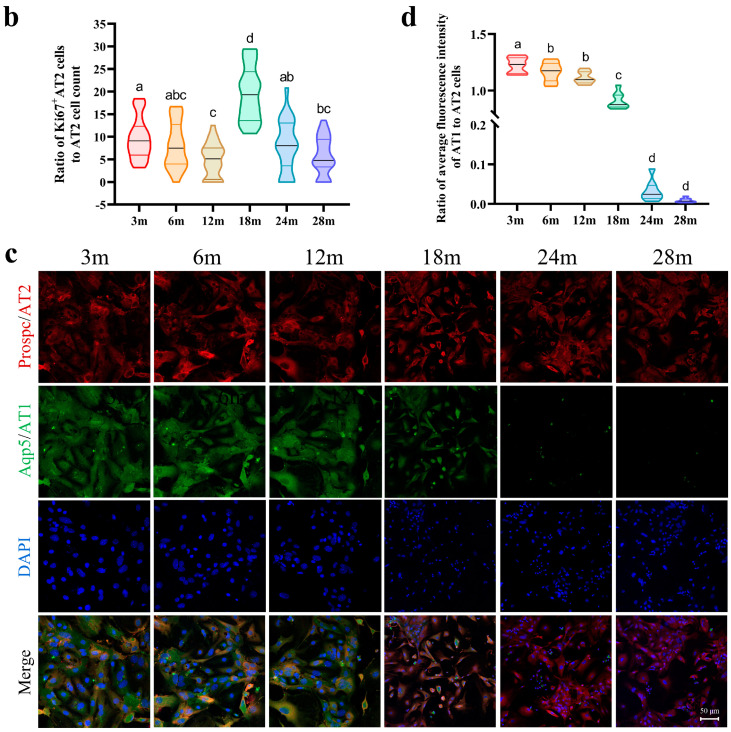
Profound declines in proliferative and differentiative capacities of AT2 cells with aging. (**a**) Immunofluorescence staining of Ki67^+^ AT2 cells in mouse lung tissues (objective: ZEISS Plan-Apo 20X, 0.8NA). (**b**) Proportion of Ki67^+^ AT2 cells relative to total AT2 cell population. Each age group had n = 3 mice, with 8 fields quantified per tissue section. (**c**) Immunofluorescence staining of primary AT2 cells at different ages after 7-day ex vivo culture to assess their potential for differentiation into AT1 cells (objective: ZEISS Plan-Apo 20X, 0.8NA). (**d**) Violin plots representing the ratio of AT1/AT2 fluorescence intensity as an indicator of AT2 cell differentiation capacity. Eight random fields were analyzed in each group. Results are presented as violin plots, with lowercase letters indicating significant differences (*p* < 0.05) between groups.

To assess changes in the proliferative capacity of AT2 cells during the aging process, we performed immunofluorescence staining on lung tissues. As depicted in Figure 2a,b, the proportion of Ki67^+^ AT2 cells exhibited a decreasing trend from 3 m to 12 m. In the 3 to 6 m period, AT2 cells undergo proliferation to accommodate the demand for alveolar amplification. From 6 to 12 m, lung tissue development transitions to the main feature of alveolar enlargement. Interestingly, we observed a temporary rise in the proportion of Ki67^+^AT2 cells at 18 m, indicating that AT2 cells attempt to promote alveolar regeneration through self-proliferation. Subsequently, from 18 to 28 m, there was a consistent decline in AT2 cell proliferation, suggesting a notable reduction in their regenerative capacity in aging lung tissue.

The differentiation capacity of AT2 cells plays a critical role in the restoration of the alveolar epithelial layer. To investigate alterations in this differentiation ability during the aging process, we extracted and cultured primary AT2 cells in vitro. Figure 2c demonstrates that throughout the process of AT2 to AT1 differentiation, the expression of Prospc gradually decreased in the AT2 cells simultaneous to the initiation of Aqp5 expression. Co-staining these markers confirmed that the cells were differentiating and expressing proteins characteristic of both AT1 and AT2 cells. On the 7th day, the highest number of AT1 cells derived from 3 m and 6 m AT2 cells was observed, forming a continuous patch of green fluorescence. By contrast, in the 18 m age group, the distribution of AT1 cells appeared more sparse, and only a limited number of AT2 cells retained their differentiation capacity in the 24 m and 28 m age groups. The number of AT2 cells differentiating into AT1 cells significantly varied across different age groups, exhibiting an age-dependent decline (Figure 2d). Notably, a significant reduction was observed at 24 m and 28 m, indicating a pronounced decrease in the differentiation ability of aging AT2 cells. Moreover, microscopy showed that younger cells had larger nuclei than older cells, with 18 months being a significant age threshold (Figure 2c). This can be attributed to the higher metabolic activity of younger cells, which require more protein and RNA synthesis for growth and differentiation, compared to aging cells that have a slower metabolism, leading to reduced functionality and an impaired ability to synthesize biological molecules. Additionally, older cells may be more susceptible to apoptosis, resulting in a smaller nucleus size.

### 2.4. Inflammatory Signaling in AT2 Cells Was Activated during Pulmonary Aging Process

**Figure 3:**
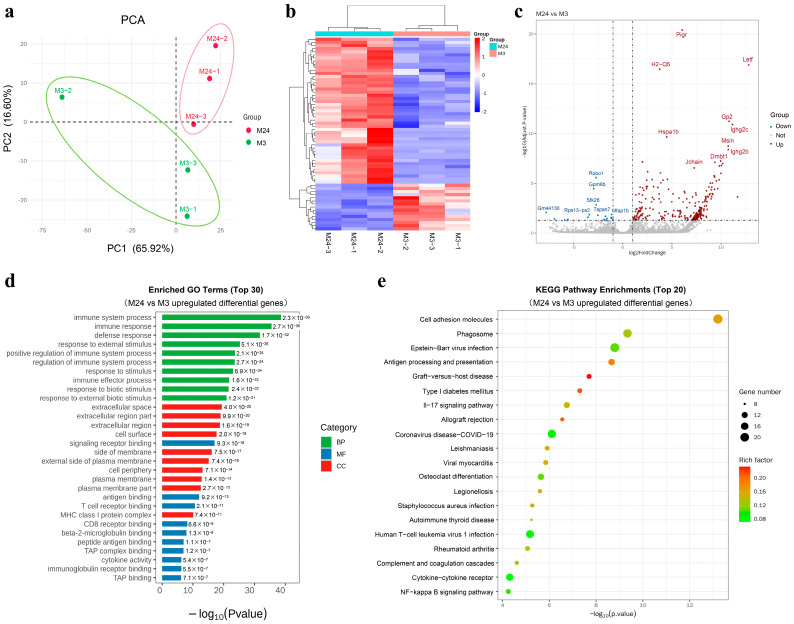
Inflammatory signaling in AT2 cells was activated during pulmonary aging process. (**a**) Transcriptomic gene expression profiles of primary AT2 cells from 3 m and 24 m mice were effectively differentiated through PCA analysis; (**b**) hierarchical clustering analysis of differentially expressed genes in 3 m and 24 m AT2 cells; (**c**) volcano plot illustrates the distribution of differentially expressed genes, their upregulation/downregulation, and the significance of differences in 3 m and 24 m AT2 cells; (**d**) GO functional enrichment analysis of differentially expressed genes in 3 m and 24 m AT2 cells. Top 30 significantly enriched functional terms sorted by *p*-value are displayed; (**e**) KEGG pathway enrichment analysis of differentially expressed genes, showing top 20 significantly enriched pathway terms.

To investigate the factors affecting AT2 function during pulmonary aging, we carried out the flow cytometry-based isolation of primary AT2 cells from 3 m and 24 m mice, followed by a transcriptomic analysis. A PCA was conducted on the transcriptome data of AT2 cells from both age groups. As illustrated in Figure 3a, both PC1 and PC2 contributed cumulatively to 82.52% of the total variance, indicating that they encapsulate the majority of sample information. Moreover, the non-overlapping distribution of the data points corresponding to the two groups in the PCA plot suggests distinct gene expression patterns that can be effectively distinguished. Subsequently, a clustered heatmap was generated to visualize the gene expression profiles of the 3 m and 24 m AT2 cells (Figure 3b). The conspicuous partition of gene expression patterns between the two groups underscores significant disparities in the gene expression profiles of AT2 cells from young and aged mice.

Subsequently, we utilized volcano plots (Figure 3c) to demonstrate the distribution and significance of differential genes between the 3 m and 24 m AT2 cells, as well as the upregulation and downregulation of gene expression levels. Our results revealed significant differences in the expression of multiple genes between the two sample groups, with 259 differential genes upregulated and 19 differential genes downregulated in the 24 m AT2 samples compared to the 3 m samples. We further interpreted our findings by exploring the functional roles of the differentially expressed genes. Notably, the upregulation of genes including *Pigr*, *Ltf*, *H2-Q5*, *Gp2*, *Ighg2c*, *Ighg2b*, *Jchain*, and *Dmbt1* suggests an enhanced immune regulatory function of AT2 cells during aging. These genes are likely involved in the immune response, inflammatory reactions, and infection resistance, aiming to protect the lungs from pathogenic invasion. Additionally, *Hspa1b* and *Dmbt1* may participate in the cellular stress response, protein folding, and cell adhesion, which can help AT2 cells address immune challenges and cellular damage, maintaining normal cell function.

Based on the transcriptomic sequencing results, we further conducted Gene Ontology (GO) annotation and functional enrichment analyses (Figure 3d). Specifically, the upregulated GO functions observed in the 24 m mice compared to their 3 m counterparts primarily related to the immune response and inflammatory response, indicating the vital role played by AT2 cells in combating pathogen invasion and modulating inflammatory reactions, which corresponds to the diminished immune system function and heightened inflammation levels observed in aged mice.

Following a Kyoto Encyclopedia of Genes and Genomes (KEGG) enrichment analysis, we pinpointed the significantly age-affected signaling pathways. The top 20 enriched pathway entries were selected for depiction in Figure 3e. Notably, the upregulated KEGG enriched pathways in the 24 m mice compared to the 3 m mice include cell adhesion molecules, potentially associated with the immune function decline and alterations in tissue repair processes observed in aged mice. Another upregulated pathway, phagosome, indicates increased phagocytosis by AT2 cells in the 24 m mice, possibly due to heightened infection risk and a stronger immune response against external pathogens. Furthermore, antigen processing and presentation, Epstein–Barr virus infection, graft-versus-host disease, the IL-17 signaling pathway, and cytokine–cytokine receptor interactions were implicated, implying an immunological imbalance and the aberrant activation of inflammatory reactions in aged mice. These findings further substantiate the characteristic decline in immune function, weakened antigen recognition ability, and heightened vulnerability to infections observed in aged mice.

### 2.5. A Correlation Was Elucidated between Proliferation, Differentiation-Regulating Genes, and Inflammatory Factors in AT2 Cells

**Figure 4:**
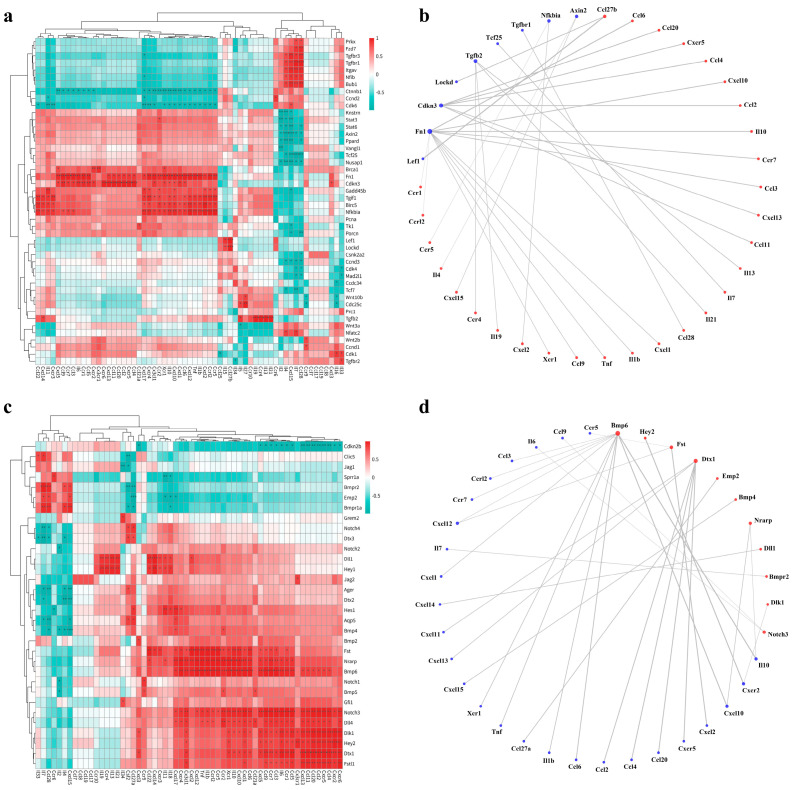
Correlation analysis between proliferation- and differentiation-regulating genes in AT2 cells and inflammatory factors. Key regulatory relationships were identified by calculating the Pearson correlation coefficient with a filtering condition of |r| > 0.8 and *p*-value <0.05 for the correlation test. Filtered results, highlighted with significance levels (* for *p*-value <0.05, ** for *p*-value < 0.01, and *** for *p*-value < 0.001), are presented in figures. Heatmap (**a**) and circular network diagram (**b**) of correlations between AT2 cell proliferation-associated genes and inflammatory factors. Heatmap (**c**) and circular network diagram (**d**) of correlations between AT2 cell differentiation-associated genes and inflammatory factors.

During AT2 cell proliferation, the activation of the Wnt/β-catenin signaling pathway and alterations in the cell cycle occur [17,18]. Previous studies have shown the impact of inflammation on cell proliferation [19,20,21]. Our preliminary research revealed a remarkable upregulation of inflammatory signals in AT2 cells from aging lungs, accompanied by a notable decrease in proliferative capacity. The decline in AT2 cell proliferative capacity is a crucial factor influencing alveolar epithelial regeneration. Therefore, we performed a correlation analysis utilizing correlation heatmaps and network diagrams to investigate the association between genes involved in AT2 cell proliferation and inflammatory genes, aiming to unravel the underlying causes and mechanisms behind the decline in AT2 cell proliferative capacity in aging lungs. Firstly, we examined the relationship between the Wnt pathway and inflammation. As depicted in Figure 4a, several genes involved in Wnt pathway transcriptional regulation, notably *Ctnnb1*, *Axin2*, *Wnt3a*, *Porcn*, *Prkx*, *Fzd7*, *Tcf7*, and *Tcf25*, exhibited a pronounced negative correlation with various inflammatory factors. This intriguing finding suggests that chronic inflammation, a hallmark of lung aging, disrupts the intricate signaling cascade of the Wnt pathway, thereby compromising the proliferative capacity of AT2 cells. To delve deeper into this phenomenon, a meticulous analysis investigating cell cycle dynamics in AT2 cells was subsequently conducted. Previous studies have reported that TGF-β acts as a pivotal signal that inhibits AT2 cell proliferation, specifically in cells experiencing cell cycle arrest [22]. Figure 4a shows a significant positive correlation between inflammatory factors and genes crucial for the TGF-β pathway, including *Tgfb2*, *Tgfbr1*, *Tgfbr3*, *Nfkbia*, *Fn1*, *Gadd45b*, *Tgif1*, *Nfib*, and *Itgav*. This observation suggests that the inflammatory milieu can continuously activate the transmission of the TGF-β pathway, resulting in the stalling of the cell cycle of AT2 cells at the G1 phase and impeding subsequent mitosis.

Subsequently, we conducted a comprehensive analysis of the correlation between genes associated with AT2 cell differentiation and inflammatory factors. Emerging evidence highlights the intricate regulation of AT2-to-AT1 cell differentiation during alveolar regeneration via multiple signaling pathways mediated by NOTCH, BMP, and YAP [9,12,23,24]. Notably, previous studies have shown that bacteria-induced alveolar injury transiently activates NOTCH signaling in AT2 cells, followed by the upregulation of the Dlk1 ligand to suppress Notch signaling, enabling AT2-to-AT1 differentiation [25]. Moreover, an interesting study has unveiled that enhanced BMP/SMAD signaling promotes AT2-to-AT1 cell differentiation, thereby facilitating the repair of a damaged alveolar epithelium [26]. As delineated in Figure 4c, the constituents of the NOTCH signaling pathway, encompassing the *Notch3* receptor, the target genes *Hey1*, *Hey2*, and *Hes1*, the ligands *Jag2*, *Dll1*, and *Dll4*, along with the pertinent regulatory proteins *Dtx1*, *Dlk1*, and *Nrarp*, all manifest notably positive associations with the majority of inflammatory factors. Moreover, Figure 4d distinctly illustrates a significant correlation between the genes *Hey2*, *Dtx1*, *Nrarp*, *Dll1*, and *Dlk1* within the NOTCH pathway and inflammatory factors. These findings signify that throughout the aging process, persistent inflammation continuously upregulates the transmission of NOTCH signaling, consequently impeding the differentiation of AT2 cells. Moreover, an analysis of Figure 4c reveals intriguing associations within the BMP pathway. Specifically, *Bmp4* displayed a significant negative correlation with *Il2, Il4, Ccl28, and Cxcl15.* Likewise, the receptors *Bmpr1a* and *Bmpr2* exhibited a negative correlation with the majority of inflammatory factors. These findings suggest that during the aging process, a heightened inflammatory response may attenuate the activity of the BMP signaling pathway. In parallel, *Fst* and *Fstl1*, acting as inhibitory proteins within the BMP pathway, exert a noteworthy positive correlation with the majority of inflammatory factors, further emphasizing the suppressive impact of the inflammatory milieu on BMP signaling. However, it is worth noting that the upregulation of *Bmp6* in the context of inflammation may represent a compensatory mechanism aimed at bolstering BMP signaling activity. Consistent with this, Figure 4d illustrates compelling correlations between *Bmp4*, *Bmp6*, *Bmpr2*, and *Fst* and inflammatory factors within the BMP pathway.

### 2.6. D-Gal-Induced Aging of A549 Cells Exhibited Inflammatory Response

**Figure 5:**
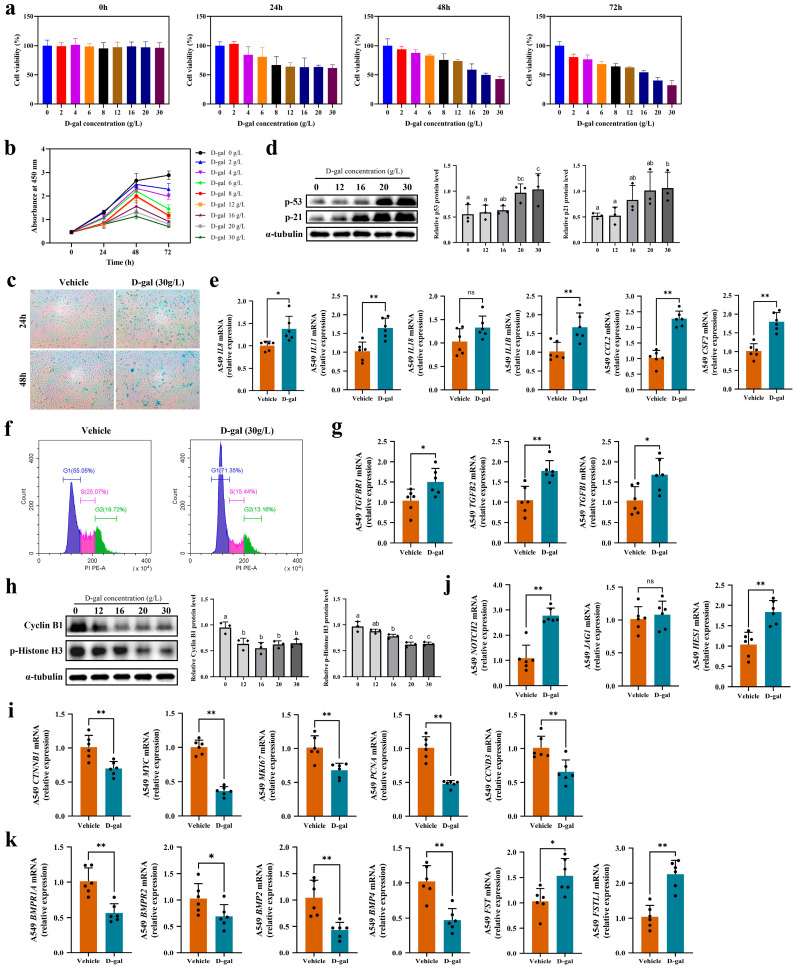
Reduced proliferation and differentiation capacities of D-gal-induced senescent A549 cells. (**a**,**b**) Cell viability of A549 cells decreased following treatment with D-gal; (**c**) β-galactosidase staining reveals cellular senescence levels in D-gal-stimulated A549 cells (n = 3); (**d**) Western blot analysis of senescence-associated proteins in A549 cells treated with D-gal for 48 h (n = 3), normalized to α-Tubulin loading control. Significant group differences were indicated by lowercase letters (*p* < 0.05); (**e**) the expression levels of inflammatory factors in A549 cells induced with 30 g/L D-gal for 48 h (n = 6); (**f**) flow cytometry analysis of cell cycle in A549 cells treated with 30 g/L D-gal for 48 h (n = 3); (**g**) mRNA levels of TGF-β pathway-related genes in A549 cells treated with 30 g/L D-gal for 48 h (n = 6); (**h**) Western blot analysis of cell cycle-associated proteins in A549 cells treated with D-gal for 48 h (n = 3), normalized to α-Tubulin loading control. Significant group differences were indicated by lowercase letters (*p* < 0.05); gene expression levels associated with proliferation (**i**) and differentiation (**j**,**k**) in A549 cells treated with 30 g/L D-gal for 48 h (n = 6); (**j**) mRNA expression levels of Notch-pathway-related genes; (**k**) mRNA expression levels of BMP-pathway-related genes. Each group consisted of six biological replicates and three technical replicates in RT-qPCR experiments. Results are presented as mean ± SD values. * *p* < 0.05; ** *p* < 0.01.

Isolating AT2 cells from aged mouse lungs proved challenging due to low yields, poor ex vivo viability, and an unstable phenotype, limiting our access to sufficient primary AT2 cells for further study. The A549 cell line serves as a valuable in vitro model for studying the metabolism and functions of AT2 cells as it effectively maintains their typical biology and characteristics [27]. Therefore, we utilized the widely-recognized A549 cell line as a substitute for primary AT2 cells to construct an in vitro aging model and further validated our transcriptomic experimental results. To determine the optimal concentration and duration of D-gal treatment for constructing an aging model using A549 cells, we initially evaluated cell viability using a CCK8 assay. As depicted in Figure 5a,b, the viability of A549 cells significantly declined with increasing concentrations and durations of D-gal treatment. After 48 h of drug exposure, A549 cell viability was notably suppressed. Subsequently, we conducted β-galactosidase staining and observed that under the same drug concentration of 30 g/L, the capacity to induce senescence was more pronounced at 48 h compared to 24 h (Figure 5c). Furthermore, we examined the expression levels of proteins associated with aging (Figure 5d). Following 48 h of D-gal treatment, the expression levels of the p53 and p21 proteins exhibited dose-dependent increases, with the highest levels observed at a concentration of 30 g/L. Consequently, we determined that a concentration of 30 g/L and a duration of 48 h are the optimal conditions for inducing senescence.

Subsequently, we evaluated the expression levels of typical inflammatory factors. As depicted in Figure 5e, A549 cells experiencing D-gal-induced aging exhibited upregulation of *IL8* (37.0%), *IL11* (60.5%), *IL18* (29.4%), *IL11B* (63.4%), *CCL2* (121.7%), and *CSF2* (77.3%). Our findings further verified the activation of an inflammatory response in epithelial cells during lung aging. The accumulation of senescent epithelial cells triggers disruptions in the overall microenvironment via the secretion of SASP and pro-inflammatory mediators, ultimately resulting in chronic inflammation.

### 2.7. Impaired Proliferation and Differentiation Capacities in D-Gal-Induced Aging of A549 Cells

To validate the decreased proliferation capacity of A549 cells under inflammatory conditions, we initially assessed changes in their cell cycle via flow cytometry. Our findings demonstrate that after 48 h of culturing A549 cells in a medium containing 30 g/L of D-gal, there was a remarkable increase of 16.3% in the proportion of cells in the G1 phase compared to the untreated group. Conversely, the proportion of cells in the S phase decreased by 9.63%, and the proportion in the G2+M phase decreased by 6.56% (Figure 5f). Notably, aged A549 cells exhibited arrest in the G1 phase. We then investigated the expression levels of crucial genes in the TGF-β signaling pathway within aged A549 cells. Our findings revealed significant increases in the expression levels of *TGFBR1* (44.5%), *TGFB2* (69.1%), and *TGFBI* (60.5%) (Figure 5g), once again substantiating the occurrence of cell cycle arrest in alveolar epithelial cells following aging. Additionally, we observed a significant downregulation of proliferation-associated proteins, Cyclin B1 and p-Histone H3, in aging A549 cells (Figure 5h). Furthermore, our findings unveiled a notable downregulation in the expression of the key protein *CTNNB1* (31.0%) and its downstream effectors *MYC* (64.0%), *MKI67* (33.0%), *PCNA* (52.7%), and *CCND3* (35.2%) along the Wnt/β-catenin pathway in senescent A549 cells (Figure 5i). The secretion of senescence-associated pro-inflammatory mediators during aging could impact the robustness of the Wnt pathway, imposing constraints on signal transduction and aggravating the decline in stem cell activity.

Next, in order to further validate alterations in the differentiation capacity of epithelial cells in the context of age-related chronic inflammation, we conducted investigations on relevant proteins in the differentiation regulatory pathway. Our results revealed a persistent activation of the Notch pathway in senescent A549 cells, accompanied by a notable increase in both the receptor *NOTCH2* and target gene *HES1* expression levels by 156.4% and 76.5%, respectively (Figure 5j). This heightened Notch signal restrains the differentiation of epithelial cells. Additionally, we found that the expression levels of the BMP signaling receptors *BMPR1A* (44.1%) and *BMPR2* (32.7%) and the ligands *BMP2* (58.5%) and *BMP4* (84.1%) were substantially downregulated in senescent A549 cells (Figure 5k). The antagonist proteins *FST* and *FSTL1* exhibited pronounced upregulation by 49.1% and 116.8%, respectively, thereby hindering the smooth transduction of the BMP signaling pathway and impeding epithelial cell differentiation progression.

In conclusion, our validation reveals that inflammation during the aging process negatively impacts the proliferation and differentiation capabilities of lung epithelial cells, thereby affecting the regeneration of epithelial structures.

## 3. Discussion

The human alveolar epithelium serves as a critical immune interface and is highly susceptible to damage from external stimuli. AT2 cells play a key role in protecting the lungs against pathogen invasion and host defense, repairing damaged alveoli, and restoring pulmonary homeostasis. Our research findings demonstrate that the alveolar epithelium is highly susceptible to damage as a consequence of aging. During lung aging, there is an age-dependent decline in the quantity of AT2 cells accompanied by structural damage and significant impairments in proliferative and differentiation capabilities. Damaged AT2 cells can activate and recruit immune cells into the alveoli, releasing a large amount of cytokines, thereby rapidly altering the immune microenvironment within the lungs [28,29]. It is worth noting that AT2 cells are critical executors responsible for epithelial regeneration. AT1 cells, which are responsible for gas exchange, cannot repair themselves once damaged and rely on differentiation from AT2 cells. The depletion and dysfunction of AT2 stem cells caused by aging can directly impact the regeneration of epithelial structures and the repair of alveoli. Furthermore, our findings demonstrate pronounced inflammatory infiltrates in aged lung tissue and the abnormal activation of inflammatory signals in AT2 cells, consistent with previous research [5,30,31,32,33]. The secretion of SASP and pro-inflammatory mediators leads to the formation of an inflammatory microenvironment. Inflammation serves as a beneficial immune response. Nevertheless, as individuals age, their tolerance to antigens and physical, chemical, and other stimuli decreases. Consequently, inflammation may transition to a chronic state of low intensity, resulting in lung tissue dysfunction and cellular degeneration [34,35].

The impact of inflammation on the proliferative and differentiation capabilities of AT2 cells exhibits a dual role, with these regulatory behaviors dependent on the type and severity of injury and the inflammatory state. The literature has reported that in the alveolar region, inflammatory responses can regulate the regenerative niche, promoting alveolar regeneration. Organoid screening experiments have revealed that inflammatory cytokines, such as IL-1 and TNF-α, can promote the proliferation of surviving AT2 cells through the IL-1/TNFα-NF-κB signaling axis while preserving their ability to differentiate, therefore promoting lung regeneration after AT2 cell injury induced by influenza [28]. In the context of chronic lung inflammation induced by cigarette smoke, AT2 cells exhibit a dynamic activation of their stem cell properties, thereby facilitating accelerated proliferation, enhanced differentiation, and augmented resistance to apoptosis [36]. However, our transcriptomic data revealed a significant negative correlation between inflammatory factors and genes associated with AT2 cell proliferation and differentiation. This suggests that the senescence-mediated inflammatory microenvironment directly inhibits the proliferative and differentiating behavior of AT2 cells. Existing research data also support this finding. During lung regeneration following injury, chronic inflammation mediated by IL-1β hinders the transition of AT2 cells into mature AT1 cells, leading to an abnormal accumulation of the transition-state DATP subpopulation and impaired alveolar regeneration [19]. During pulmonary inflammation, activated neutrophils have been shown to hinder the proliferation of AT2 cells and induce their apoptosis [37]. Similarly, studies have found that blunt chest trauma can activate alveolar macrophages and recruit neutrophils to the alveolar region, triggering an inflammatory response and inducing apoptosis in AT2 cells, further exacerbating the detrimental effects of inflammation on lung tissue [38]. An inflammatory response may activate signaling pathways related to inflammation, including the NF-κB pathway, which alters gene expression patterns and regulatory networks in stem cells [39]. The upregulation of specific inflammatory genes, like miR-155, might further exacerbate the depletion of stem cell populations [20]. Inflammatory mediators such as TNF-α and IL-1β have been demonstrated to possess the capacity to impede cell proliferation and induce apoptosis across multiple cell types [21,40]. The maintenance of pulmonary immune homeostasis requires the coordinated efforts of lung epithelial cells, local immune cells derived from the bone marrow and lymphatic system, and recruited immune cells [41,42]. In aged lung tissues, the disruption of pulmonary immune homeostasis leads to the recruitment of numerous inflammatory factors to the alveolar region. Persistent and prolonged chronic inflammation continually stimulates AT2 cells, culminating in irreversible cellular damage. Additionally, the inhibition of regulatory pathways involved in differentiation impedes the successful transition of AT2 cells into mature AT1 cells, hampering the restoration of a damaged alveolar epithelium and subsequently affecting pulmonary gas exchange. The inflammatory microenvironment induced by aging hinders alveolar regeneration.

In our correlation analysis, we found a significant positive correlation between cell cycle arrest-related genes (such as *Tgfb2*, *Tgfbr1*, *Tgfbr3*, and *Tgif1*) and inflammatory factors. This indicates that inflammatory factors could hinder the cell cycle of AT2 cells, thus affecting their proliferative capacity. Through further meticulous analyses, we unearthed a compelling negative correlation between key cell cycle regulatory genes, namely *Cdk4*, *Cdk6*, and *Ccnd3*, and inflammatory factors. The proteins encoded by *Cdk4* and *Cdk6* play a pivotal role in facilitating the transition from the G1 to the S phase of the cell cycle by forming complexes with *Ccnd3*. Additionally, our research has yielded significant evidence of a positive correlation between the *Cdkn3* gene, which encodes the CDK inhibitor, and the majority of inflammatory factors. This inhibition effectively hampers the activity of CDK-related proteins and disrupts cell cycle progression. Furthermore, the protein encoded by Ppard exerts a pivotal influence on cellular proliferation by actively participating in the intricate regulation of cellular metabolism and growth, which displays a significant negative correlation with key inflammatory factors such as *Il2*, *Il4*, *Cxcl15*, and *Ccl28*. Moreover, we discovered a noteworthy negative association between genes involved in spindle formation and chromosome segregation, including *Prc1*, *Knstrn*, *Mad2l1*, *Nusap1*, and *Bub1*, and inflammatory factors. Remarkably, these genes are downregulated in the inflammatory conditions induced by aging, potentially compromising the precise orchestration of mitosis. Intriguingly, we observed significant alterations in transcriptional regulation-related genes during the aging process. In particular, *Brca1*, which encodes a protein involved in DNA double-strand damage repair throughout the cell cycle; *Birc5*, which encodes a protein essential for preventing abnormal cell death during cell division; and *Lockd*, which is responsible for cellular stress response and immune regulation, all exhibited a notable positive correlation with inflammatory factors. Additionally, the proteins encoded by the genes *Stat3* and *Stat6* are crucial in mediating the signal transduction of various cytokines, thereby regulating cellular immune responses and proliferation. Remarkably, these two genes exhibit positive correlations with most inflammatory factors. The upregulation of these genes further supports the notion that age-related chronic inflammation leads to cellular damage and apoptosis, triggering the activation of defense genes for intracellular regulation. In conclusion, these data indicate that the inflammatory response triggered by aging significantly arrested the cell cycle of AT2 cells, impairing their proliferative capacity and thereby impacting alveolar regeneration.

This study presents a systematic elucidation of age-related structural, quantitative, and functional alterations within the mouse alveolar epithelium, with a particular focus on how inflammatory stimuli guide the fate behavior of AT2 stem cells during lung injury repair subsequent to physiological aging. These findings offer new insights into how chronic inflammation impairs stem cell-mediated alveolar regeneration. Furthermore, we are collectively working toward a more comprehensive exploration of the functional characteristics of AT2 cells in response to inflammation, intending to fully elucidate and delve deeper into the specific regulatory mechanisms underlying this physiological correlation. In the future, there is a promising prospect to utilize systemic inflammation modulation strategies to promote alveolar regeneration and delay or even reverse age-related pulmonary diseases, thereby reducing their incidence and mortality rates among the elderly population.

## 4. Materials and Methods

### 4.1. Mouse and Tissue Sampling

All animal experiments were conducted in accordance with guidelines prepared by an animal welfare organization and approved by the Animal Care and Use Committee of China Agricultural University (approval number AW41203202-5-3). Pathogen-free C57BL/6J mice were obtained from Beijing Vital River Laboratory Animal Technology Co., Ltd., Beijing, China and housed individually in a controlled environment (temperature, 22–24 °C; humidity, 60%) with ad libitum access to food and water. Six age groups of mice were included in this study, ranging from 3 to 28 months old (including 3 m, 6 m, 12 m, 18, 24 m, and 28 m), and all mice underwent natural aging without any exogenous stimuli or interventions.

After anesthetizing the mice, lung tissues were harvested by perfusing physiological saline through the heart to remove blood and turn the tissues white. Subsequently, 4% paraformaldehyde (PFA, G1101, Servicebio, Wuhan, China) was perfused for the fixation of the organs and tissues. The lung lobes were dissected, fixed in PFA for 48 h, embedded in paraffin, and sectioned into 5 μm slices. Lung tissues that were not perfused with PFA were rapidly frozen in liquid nitrogen and stored at −80 °C for subsequent protein and RNA analyses.

### 4.2. HE Staining

The structure of the alveolar epithelium of the lung tissues was assessed using an HE staining kit (G1120, Solarbio, Beijing, China). The lung tissue sections were subjected to staining according to the manufacturer’s instructions. Each age group consisted of six biological replicates. Finally, the sections were examined under a bright-field microscope (DM6B, Leica, Wetzlar, Germany) using a 20× objective lens.

### 4.3. Immunofluorescent Staining

Tissue sections embedded in paraffin were subjected to deparaffinization and hydration, followed by immersion in sodium citrate antigen retrieval solution (C1031, Solarbio, Beijing, China) and microwave boiling. Afterward, the sections were immersed in a PBS solution containing 1% Triton X-100 (T8200, Solarbio, Beijing, China) for 15 min and blocked with 10% goat serum (C01-03001, Bioss, Beijing, China) at room temperature for 1 h. Primary antibodies were added and incubated overnight at 4 °C. The following day, the sections were incubated with secondary antibodies at room temperature in the dark for 1 h (detailed antibody information is shown in Supplementary Table S1). The sections were then stained with a DAPI working solution (C0065, Solarbio, Beijing, China) for 10 min and mounted using an anti-fade mounting medium (P0126, Beyotime, Shanghai, Beijing). Observation and photography were performed under a laser confocal microscope (LSM 900/Axio Observer 7, ZEISS, Oberkochen, Germany). Cells grown on glass coverslips were stained using the same method.

### 4.4. Transmission Electron Microscope (TEM) Detection

Fresh lung tissue samples, measuring approximately 1 mm^3^ in size, were fixed overnight at 4 °C using a 2.5% glutaraldehyde (P1126, Solarbio, Beijing, China) fixative. Subsequently, the samples were fixed with a 1% osmium tetroxide solution for 2 h. Dehydration was performed using a gradient ethanol series (50%, 70%, 80%, 90%, and 95% for 15 min each), followed by two treatments with pure acetone for 20 min each. The samples were then infiltrated with a mixture of embedding resin and acetone (*v*/*v* = 1/1) for 2 h, followed by a mixture of embedding resin and acetone (*v*/*v* = 3/1) for 3 h, and finally left overnight in pure embedding resin. The processed samples were embedded, polymerized, and sectioned into ultra-thin slices (70–100 nm). These sections were stained with 2% uranyl acetate and lead citrate for 5–15 min each and observed under a transmission electron microscope (H-7650, Hitachi, Tokyo, Japan). Each group comprised three biological replicates.

### 4.5. Cell Culture

Human A549 cells were obtained from Procell Life Science & Technology Co., Ltd. (Wuhan, China) and were cultured in Ham’s F-12K medium (PM150910, Procell, Wuhan, China) supplemented with 10% fetal bovine serum (164210, Procell, Wuhan, China) and 1% penicillin–streptomycin (PB180120, Procell, Wuhan, China), following standard laboratory protocols. The average doubling time of the A549 cells was approximately 22 h, indicating their rapid proliferation rate.

Primary AT2 cells were cultured in a specialized medium (CM-M003, Procell, Wuhan, China) fortified with essential growth factors to support their specific requirements. Furthermore, the culture vessels used for the primary AT2 cells were carefully coated with a layer of 5 μg/cm^2^ of mouse tail collagen (354236, Corning, Bedford, MA, USA) to enhance cell adhesion. All cell cultures were maintained in a dedicated incubator (HERAcell^TM^ 150i, Thermo Scientific, Waltham, MA, USA) set at a controlled temperature of 37 °C with a 5% CO_2_ atmosphere.

### 4.6. Cellular Senescence Induction

In order to prevent cellular replicative senescence, we utilized low-passage proliferative A549 cells (<15 passages) in this study. These cells were seeded at a density of 1.5 × 10^5^ cells per well in a 6-well plate and cultured under standard conditions for 24 h to promote firm attachment and proliferation. After this incubation period, each well in the treatment group was supplemented with 2 mL of culture medium containing D-galactose (concentrations: 0, 2, 4, 6, 8, 12, 16, 20, and 30 g/L) for a specified duration (e.g., 24, 48, 72 h), in order to induce cellular senescence. Subsequent staining procedures, as well as extractions of RNA and proteins, were performed on these cells for further comprehensive analysis.

### 4.7. Cell Viability Testing

In this experiment, we utilized a Cell Counting Kit-8 (CCK-8, CK04, Dojindo, Kyushu Island, Japan) according to the provided instructions. After a certain period of D-galactose-induced senescence in the A549 cells, 10 μL of CCK-8 solution was added, and the cells were incubated at 37 °C in a light-protected, constant-temperature chamber for 60 min. Absorbance at 450 nm was measured using a multi-mode microplate reader (SYNERGY^TM^ HTX, BioTek Instruments, Inc., Winooski, VT, USA). Cell viability (%) was calculated as [(%Treatment − %Blank)/(%Control − %Blank)] × 100%. Each group comprised 6 technical replicates.

### 4.8. β-Galactosidase Staining

In this study, senescent cells were identified utilizing a commercially available β-galactosidase staining kit (G1580, Solarbio, Beijing, China). The specific experimental operations were carried out according to the method provided in the instructions. The stained senescent cells were meticulously observed and characterized using an inverted microscope (DMi8, Leica, Wetzlar, Germany). Each group comprised three replicates.

### 4.9. Flow Cytometric Analysis of Cell Cycle Progression

A549 cells in the logarithmic growth phase were inoculated into a 6-well plate at a density of 1.5 × 10^5^ cells per well. Following 24 h of incubation with 30 g/L of D-galactose, the cells were digested to obtain a single-cell suspension. Subsequently, 750 μL of pre-chilled absolute ethanol was gradually introduced, and the cell suspension was placed in a −20 °C freezer overnight for fixation. The next day, the cells were resuspended in 425 µL of Cell Staining Buffer (420201, Biolegend, San Diego, CA, USA), 50 μL of 100 µg/mL RNase (R1030, Solarbio, Beijing, China), as well as 25 µL of Propidium Iodide Solution (421301, Biolegend, San Diego, CA, USA) and incubated on ice in the absence of light for 15 min. Cells were acquired at a low speed, and red fluorescence (PE channel) was detected at 488 nm using a flow cytometer (CytoFLEX, BECKMAN COULTER, Suzhou, China). Each group comprised three replicates.

### 4.10. Isolation and Culture of AT2 Cells

To prepare a mixture of enzymes for lung tissue digestion, 780 U of Type I collagenase (260 U/mg, 17100017, Gibco, Grand Island, NY, USA), 40 U of Elastase (≥3 U/mg, LS002294, Worthington, Lakewood, CO, USA), and 20 U of Dispase II (0.9 U/mg, 4942078001, Roach, Mannheim, Germany) were dissolved in a total volume of 10 mL of PBS (FB13356, FEIMOBIO, Beijing, China) supplemented with Ca^2+^ and Mg^2+^.

The mice were anesthetized, and their lungs were rinsed with a physiological saline solution through cardiac perfusion to remove blood. Following tracheal intubation, the lungs were sequentially injected with 1.0 mL of enzyme solution and 0.6 mL of 1% low-melting-point agarose. The lungs were then removed and placed in the remaining enzyme solution for digestion at 37 °C for approximately 40 min. After digestion, the cell suspension was centrifuged, resuspended in 5 mL of red blood cell lysis buffer (Solarbio, R1010), and incubated on ice for 10 min. Subsequently, the suspension was filtered through a 40 μm cell strainer (SORFA, 251100). The cells were cultured at 37 °C with 5% CO_2_ for 45 min and then transferred to a new culture dish. This process was repeated three times to eliminate most fibroblasts. Finally, the cell suspension was transferred to a 25T culture flask coated with 5 μg/cm^2^ of mouse tail collagen (Corning, 354236) and cultured for 24 h before medium replacement.

### 4.11. Flow Cytometry Analysis and Sorting of AT2 Cells

Lung tissues from 3 m and 24 m mice were enzymatically digested to produce lung cell suspensions, followed by subsequent staining procedures. Within a 500 μL volume of cell suspension, 1 μL of FITC rat anti-mouse CD45 antibody (BD, 553079), PE/Cyanine 7 anti-mouse CD31 antibody (102418, Biolegend, San Diego, CA, USA), APC CD326 (EpCAM) monoclonal antibody (17-5791-80, Invitrogen, Carlsbad, CA, USA), and APC/Cyanine 7 anti-mouse MHCII (I-A/I-E) antibody (107627, Biolegend, San Diego, CA, USA) were added. The cells were then incubated on ice in the absence of light for 30 min for optimal labeling. Subsequently, 5 μL of 7-AAD viability staining solution (420404, Biolegend, San Diego, CA, USA) was added to facilitate the selection of viable cells. Ultimately, the cells underwent a flow cytometry analysis (FACSAria^TM^ III Cell Sorter, BD, Franklin Lake, NJ, USA) to identify and isolate CD45^−^ CD31^−^ CD326^+^ MHCII^+^ cells, specifically sorted as AT2 cells.

### 4.12. RNA Extraction and RT-qPCR

The RNA extraction process involved the lysis of the lung tissue samples and cells using pre-chilled TRNzol reagent (DP424, TIANGEN, Beijing, China) for 30 min, followed by RNA isolation as per the manufacturer’s protocol. RNA concentration was determined using a NanoDrop spectrophotometer (Thermo Scientific, Madison, WI, USA). Subsequently, cDNA synthesis was performed using the cDNA Reverse Transcription Kit (G592, abm, Zhenjiang, China). For a gene expression analysis, an RT-qPCR was conducted on a real-time PCR instrument (QuantStudioTM 5, Thermo Scientific, Marsiling Industrial Estate, Singapore), using SYBR Green (A25742, Thermo Scientific, Madison, WI, USA) as the fluorescent dye. Each group comprised 6 biological replicates, with 3 technical replicates. The data were presented as fold change values calculated using the ∆∆Ct method, with GAPDH used as the reference gene for normalization. The primer sequences used in this study are provided in Supplementary Tables S2 and S3.

### 4.13. Western Blots

For lung tissue samples, approximately 80–100 mg of fresh tissue was incubated on ice for 30 min with 1 mL of lysis buffer containing 980 μL of RIPA (R0010, Solarbio, Beijing, China), 10 μL of PMSF (P0100, Solarbio, Beijing, China), and 10 μL of protein phosphatase inhibitor (P1260, Solarbio, Beijing, China) and underwent homogenization and centrifugation. For monolayer adherent cells grown in a 6-well plate, 1 mL of lysis buffer was added to each well to obtain the protein supernatant. SDS-PAGE was utilized to separate the high-molecular-weight proteins in each sample. Incubation with specific antibodies against the target proteins was performed (Detailed antibody information is shown in Supplementary Table S4), and the bound antibodies were detected and visualized using a chemiluminescence imaging system (ChemiScope 6100, Clinx Science Instruments, Shanghai, China). Each group comprised three replicates.

### 4.14. RNA Sequencing Analysis

AT2 cells were isolated from the lungs of both 3 m and 24 m mice using flow cytometry (FACSAria^TM^ III Cell Sorter, BD, Franklin Lake, NJ, USA). Three biological replicates were collected for each age group by pooling lung cells from multiple mice to ensure an adequate number of AT2 cells for subsequent sequencing. A transcriptome sequencing analysis was conducted on the Illumina NovaSeq 6000 platform (sequencing mode: PE150) at Shanghai Applied Protein Technology, Shanghai, China.

### 4.15. Statistical Analyses

The data were presented as mean ± SD values or in violin plot form, with n representing the number of experimental replicates or the number of mice. The data analysis was conducted using IBM SPSS Statistics version 26.0 software. When comparing differences between two groups, t-tests were employed to calculate *p*-values. Statistical significance was indicated by asterisks, where * *p* < 0.05 and ** *p* < 0.01, while “ns” denoted non-significance. For comparisons among multiple groups, post hoc multiple comparisons using Duncan’s analysis within a one-way ANOVA test were performed, and lowercase letters were assigned to indicate statistical differences. In all cases, *p* < 0.05 or *p* < 0.01 was considered statistically significant. Graphical representations of the analyzed data were generated using GraphPad Prism version 9.0 software.

## Data Availability

The data presented in this study are available in the article and Supplementary Materials. The transcriptomics data underlying this article can be accessed through the GEO database at https://www.ncbi.nlm.nih.gov/geo/query/acc.cgi with the accession number GSE253790, URL (accessed on 19 January 2024). For any further inquiries, please direct your questions to the corresponding author.

## Supplementary Materials

The following supporting information can be downloaded at https://www.mdpi.com/article/10.3390/ijms25063476/s1.
