# Supplementary material for: Age-Dependent Inflammatory Microenvironment Mediates Alveolar Regeneration
Source: Int J Mol Sci. 2024 Mar 20;25(6):3476. doi: 10.3390/ijms25063476 (PMC10970842; doi:10.3390/ijms25063476)
Supplement: Supplementary file 1 [file ijms-25-03476-s001.zip › ijms-2843958-supplementary.pdf]

Supplementary Materials

Table S1. Antibody information for immunofluorescence staining experiment.

Antibodies	Source	Identifier	Host
Anti-Prosurfactant protein C/Prospc antibody	abcam	ab90716	Rabbit
Aquaporin 5/Aqp5 antibody	Santa Cruz Biotechnology	sc-514022	Mouse
Recombinant Anti-Ki67 antibody	abcam	ab279653	Mouse
Alexa Fluor 488-labeled Goat Anti-Mouse IgG(H+L)	abcam	A0428	-
Alexa Fluor 647-labeled Goat Anti-Rabbit IgG(H+L)	abcam	A0468	-

Table S2. Primer sequences for mouse-specific genes used in our experiment.

Gene	Forward Primer (5′ to 3′)	Reverse Primer (5′ to 3′)
Gapdh	TGGCCTTCCGTGTTCCTAC	GAGTTGCTGTTGAAGTCGCA
Aqp5	AGAAGGAGGTGTGTTCAGTTGC	TAATGGCCGGATTGATGTGGC
Hopx	ATACTGTCCCCTCGGAGTGT	GTGCGCGTCTGACTAAGGAT
Pdpn	GTTTTGGGGAGCGTTTGGTTC	CATTAAGCCCTCCAGTAGCAC
Igfbp2	CAGACGCTACGCTGCTATCC	CCCTCAGAGTGGTCGTCATCA
Sftpb	TGAACAGGCTATGCCACAGG	GACCGCGTTCTCAGAGGTG
Sftpc	CATGGGCCTCCACATGAGTC	GCTTATAGGCCGTCAGGAGC
Sftpd	ACGTGGACTAAGTGGACCTCC	CCTTTTGCCCCTGTAGATCCTT

Table S3. Primer sequences for human-specific genes used in our experiment.

Gene	Forward Primer (5′ to 3′)	Reverse Primer (5′ to 3′)
GAPDH	GGAGCGAGATCCCTCCAAAAT	GGCTGTTGTCATACTTCTCATGG
CTNNB1	CATCTACACAGTTTGATGCTGCT	GCAGTTTTGTCAGTTCAGGGA
MYC	TTCATAACGCGCTCTCCAAGTA	TCAAGAGTCCCAGGGAGAGT
MKI67	ACGCCTGGTTACTATCAAAAGG	CAGACCCATTTACTTGTGTTGGA
PCNA	GCCTGACAAATGCTTGCTGAC	TTGATGAGGTCCTTGAGTGCC
CCND3	CCGAAACTTGGCTGAGCAGA	GTGTTTACAAAGTCCGCGCC
NOTCH2	GGTGGATACAGATGCGAGTGT	CTTAAGACAATGCCCTGGATGG
JAG1	TCACGGGAAGTGCAAGAGTC	GTTTCACAGTAGGCCCCCTC
HES1	AAGAAAGATAGCTCGCGGCA	CCTCGGTATTAACGCCCTCG
TGFBR1	ACGGCGTTACAGTGTTTCTG	GCACATACAAACGGCCTATCTC
TGFB2	GTGCTCTGTGGGTACCTTGA	GCGCTGGGTTGGAGATGTTA
TGFBI	AGGCCTTCGAGAAGATCCCT	GAGATGATCGCCTTCCCGTT
BMPR1A	CCTGTTGTCATAGGTCCGTTTT	ATCCTGTTCCAAATCACGATTGT
BMPR2	CACTCAGTCCACCTCATTCATTT	TTGTTTACGGTCTCCTGTCAAC
BMP2	TCCTAAGGAGGACGACAGCA	TGGGGTGCGCAAGTTATTCT

BMP4	CTAGGTGAGTGTGGCATCCG	ACGACCATCAGCATTCGGTT
FST	GTGTATCAAAGCAAAGTCCTGTGAA	GCTCAGGTTTTACGGGCAGA
FSTL1	ACCCACCTGTCTCTGCATTG	AGCTCATCACGGTTGGACTG
IL8	TCTGCAGCTCTGTGTGAAGG	TTCTCAGCCCTCTTCAAAAACTTC
IL11	CATGAACTGTGTTTGCCGCC	GGAATCCAGGTTGTGGTCCC
IL18	ATCGCTTCCTCTCGCAACAA	GAGGCCGATTTCCTTGGTCA
IL1B	AGCTACGAATCTCCGACCAC	CGTTATCCCATGTGTCGAAGAA
CCL2	CCTTCATTCCCCAAGGGCTC	GGTTTGCTTGTCCAGGTGGT
CSF2	GAGACACTGCTGCTGAGATGA	AGGGCAGTGCTGCTTGTAG

Table S4. Antibody information for Western blot analysis in our experiment.

Antibodies	Source	Identifier	Host	Dilution
Recombinant anti-alpha tubulin antibody	abcam	ab52866	Rabbit	1:2500
SFTPC antibody	GeneTex	GTX134340	Rabbit	1:1000
Anti-Aquaporin 5 (Aqp5) antibody	abcam	ab78486	Rabbit	1:10000
Anti-HOPX antibody	abcam	ab195974	Rabbit	1:1000
Recombinant anti-RAGE antibody	abcam	ab181293	Rabbit	1:5000
P53 Monoclonal antibody	proteintech	60283-2-Ig	Mouse	1:7000
P21 Polyclonal antibody	proteintech	27296-1-AP	Rabbit	1:600
Recombinant anti-Cyclin B1 antibody	abcam	ab181593	Rabbit	1:2000
Recombinant anti-Histone H3 (phospho S10) antibody	abcam	ab267372	Rabbit	1:1000
HRP-labeled Goat Anti-Rabbit IgG(H+L)	Beyotime	A0208	-	1:1000
HRP-labeled Goat Anti-Mouse IgG(H+L)	Beyotime	A0216	-	1:1000
